# Dental Metallurgy

**Published:** 1911-07-15

**Authors:** 


Dental Metallurgy.—This is the third edition of a most excellent manual on dental metallurgy by Ernest A. Smith, of the Royal College of Surgeons in England. Published by J. & A. Churchill, 7 Great Marlborough St., London. Mr. Smith is a teacher and worker in practical metallurgy and has succeeded in making a book for dental students that is remarkable for its concise and comprehensive treatment of the metals most used in dentistry. It is not only a good student manual, but every dental practitioner would be greatly benefited by reading it, and the making of plate alloys and solders, and refining of scrap, would become a much easier matter to most practitioners than it now is. The price is two dollars and sixty cents, and it may be ordered through the dealers.

